# Elevated FGF23 and disordered renal mineral handling with reduced bone mineralization in chronically erythropoietin over-expressing transgenic mice

**DOI:** 10.1038/s41598-019-51577-z

**Published:** 2019-10-18

**Authors:** Arezoo Daryadel, Luciano Natale, Petra Seebeck, Carla Bettoni, Udo Schnitzbauer, Max Gassmann, Carsten A. Wagner

**Affiliations:** 1 Institute of Physiology, University of Zurich, Zurich, Switzerland; 2 Zurich Center for Integrative Human Physiology (ZIHP), University of Zurich, Zurich, Switzerland; 3 National Centre for Competence in Research NCCR “Kidney.CH”, Zurich, Switzerland; 4 Zurich Integrative Rodent Physiology (ZIRP), University of Zurich, Zurich, Switzerland; 5 Institute of Veterinary Physiology, University of Zurich, Zurich, Switzerland; 6 Universidad Peruana Cayetano Heredia (UPCH), Lima, Peru

**Keywords:** Calcium and vitamin D, Kidney

## Abstract

Fibroblast Growth Factor 23 (FGF23) is a phosphaturic factor causing increased renal phosphate excretion as well as suppression of 1,25 (OH)_2_-vitamin D_3._ Highly elevated FGF23 can promote development of rickets and osteomalacia. We and others previously reported that acute application of erythropoietin (EPO) stimulates FGF23 production. Considering that EPO is clinically used as chronic treatment against anemia, we used here the Tg6 mouse model that constitutively overexpresses human EPO in an oxygen-independent manner, to examine the consequences of long-term EPO therapy on mineral and bone metabolism. Six to eight weeks old female Tg6 mice showed elevated intact and C-terminal fragment of FGF23 but normal plasma levels of PTH, calcitriol, calcium and phosphate. Renal function showed moderate alterations with higher urea and creatinine clearance and mild albuminuria. Renal phosphate excretion was normal whereas mild hypercalciuria was found. Renal expression of the key proteins TRPV5 and calbindin D28k involved in active calcium reabsorption was reduced in Tg6 mice. Plasma levels of the bone turnover marker osteocalcin were comparable between groups. However, urinary excretion of deoxypyridinoline (DPD) was lower in Tg6 mice. MicroCT analysis showed reduced total, cortical, and trabecular bone mineral density in femora from Tg6 mice. Our data reveal that chronic elevation of EPO is associated with high FGF23 levels and disturbed mineral homeostasis resulting in reduced bone mineral density. These observations imply the need to study the impact of therapeutically applied EPO on bone mineralization in patients, especially those suffering from chronic kidney disease.

## Introduction

Fibroblast growth factor (FGF23) is a bone-derived hormone that regulates renal phosphate (Pi) and calcium (Ca^2+^) handling and 1,25 (OH)_2_ vitamin D_3_ homeostasis^1,2^. FGF23 reduces via its co-receptor α-klotho renal phosphate re-absorption by down regulating the sodium phosphate co-transporter NaPi-IIa in the proximal part of nephron. Moreover, α-klotho regulates the apical entry of Ca^2+^ in the distal nephron via its interaction with TRPV5 channels^3^. Further, FGF23 suppresses circulating levels of 1, 25(OH) vitamin D_3_ by inhibiting α_1_-hydroxylase (*Cyp27b1*) and stimulating 24-hydroxylase (*Cyp24a1*) that produces and degrades 1, 25(OH)_2_ vitamin D_3_, respectively, in the kidney^4^. During chronic kidney disease (CKD) FGF23 excessively increases, while α-klotho is reduced, and hyperphosphatemia ensues^2^. Moreover, many patients with CKD develop renal anemia. Recent studies uncovered a relationship of FGF23 to erythropoiesis and anemia^5–7^ and EPO production^8–11^. FGF23 and EPO appear to be part of a regulatory loop where FGF23 can suppress EPO production whereas EPO stimulates FGF23^7,10^.

However, most evidence for the interaction between EPO and FGF23 comes from short-term *in vitro* or *in vivo* experiments studying the effects of acute application of EPO (e.g. over the range of a few hours to 3–4 days). In contrast, clinically EPO is usually given during long-term treatment (e.g. over months or even years), particularly in patients with chronic kidney disease (CKD)^12^. Thus, we examined the effects of long-term elevation of EPO levels in the transgenic mouse model Tg6 constitutively overexpressing human EPO that represents, a well established *in-vivo* model to study chronic effects of EPO^13,14^. Tg6 mice are characterized by excessive erythrocytosis resulting from a 10 to 12- fold elevation of human EPO plasma levels during the first 8 to 9 postnatal weeks^15,16^. Moreover, in elderly Tg6 mice, reduced bone density has been found which has been attributed to direct stimulatory effects of EPO on osteoclast activity^17^.

Our data demonstrate elevated FGF23 levels and disturbed mineral homeostasis paralleled by reduced bone mineral density. This implies that chronic stimulation of FGF23 by EPO is negatively impacting on mineral homeostasis and bone.

## Materials and Methods

### Experimental animals

The Tg6 mouse line was generated as previously described^13,18^. The resulting transgenic mouse line (Tg6) shows increased EPO levels in plasma and brain and was bred by mating hemizygous males to wild-type C57BL/6 females. Half of the offspring was hemizygous for the transgene while the other half was wild-type (WT) and served as a control. All experiments were performed in 6–8 weeks old females and their WT female littermates. At the beginning of the experiment, mice were transferred to individual metabolic cages (Tecniplast, Buguggiate, Italy) and fed standard diet (0.8% Pi, 1% calcium, Kliba Nafag, Augst, Switzerland) for 48 hours. The last 24 hours urine was collected under mineral oil in the urine collector. Mice were then sacrificed under isoflurane anesthesia. Upon opening the abdominal cavity, blood was collected from the vena cava and centrifuged at 4 °C in heparinised tubes for 7 minutes at 8000 rpm. Hematocrit was measured in capillaries whose walls were coated with heparin. After filling the capillary, capillaries were centrifuged in a special centrifuge (Haematokrit 210, Hettich Zentrifugen; Huber & co. AG, Reinach, Switzerland) at 8000 rpm for 5 minutes. The volumetric content of the sedimented erythrocytes could be read off a scale as the percentage of the total blood volume. Plasma and organs were snap frozen in liquid nitrogen and stored at −80 °C for further analysis. Urine was centrifuged at 10000 rpm for 10 minutes and stored at −20 °C. All experiments were performed in accordance with the Swiss and international laws of animal protection, and welfare and all protocols were approved by the appropriate local veterinary authority (Kantonales Veterinäramt Zürich).

### Plasma and urine parameters analysis

Plasma total iron, urea, creatinine, phosphate and calcium and urinary phosphate, urea, calcium and creatinine were analyzed using a UniCel® SYNCHRON® DxC 800 Synchron Clinical System (Beckman Coulter). Urine albuminuria was detected by coomassie blue SDS-Page gel loaded with urine samples (equivalent to a total of 2.5 mg creatine) and BSA (0.5 mg/ml) was loaded as a positive control.

### RNA extraction and real-time RT-PCR

All procedures were performed as previously described^19^. Briefly, kidney, tibia, liver, spleen, ileum and isolated bone marrow cells^20^ were homogenized either with QIAzol® lysis reagent (Qiagen) or RLT buffer supplemented with β-mercaptoethanol. RNA from homogenates was extracted using the Qiagen RNeasy Mini Kit (Qiagen, Hombrechtikon, Switzerland) following the protocol provided by the supplier. After RNA quantification using a Nanodrop ND-1000 spectrophotometer (Thermo Scientific), reverse transcription was carried out using the Taqman Reverse Transcription Kit (Applied Biosystems, Zug, Switzerland) according to the manufacturers protocol. To quantify relative messenger RNA (mRNA) expression, specific sets of primers and probes for mouse FGF23, Hepcidin, NaPi-IIb, VDR and Cyp27b1 (supplementary Table 1) were designed using Primer Express (Applied Biosystems) and purchased from Microsynth, (Switzerland). The specificity of all primers was tested using adult mouse kidney, liver and bone cDNA by conventional PCR. Each pair of primer resulted only in a single band of the expected size (data not shown). The probes were labelled with the reporter dye FAM at the 5′ end and the quencher dye TAMRA at the 3′ end. The complementary DNA was amplified using mouse primers listed in Supplementary (Table 1) in RT-PCR reactions using the KAPA PROBE FAST qPCR Kit Master Mix (KAPA BIOSYSTEMS, Boston USA) containing primers (5 μM) and probe (25 μM) to amplify cDNA in a 7500 Fast Real Time PCR System (Applied Biosystems, Zug, Switzerland). Each reaction was done in triplicates and the average calculated. Samples without enzyme in the RT reaction were used as negative controls to exclude contamination with genomic DNA. The cycle number at a given threshold (Ct) was measured. The expression of genes of interest were normalized either to the reference genes HPRT and ribosomal 18 s (rRNA) (Applied Biosystems), when giving comparable results, and calculated by the formula ${\rm{R}}={2}^{({{\rm{ct}}}_{{\rm{18s}}{\rm{or}}{\rm{HPRT}}}-{{\rm{Ct}}}_{{\rm{gene}}{\rm{of}}{\rm{interest}}})}.$

**Table 1:** Metabolic data.

	WT	Tg6
Body weight [g]	19.2 ± 0.5	18.9 ± 0.5
Food intake [g/24 h]	4.0 ± 0.2	3. 5 ± 0.2
Water intake [ml/24 h]	5.0 ± 0.2	6.7 ± 0.5*
Feces weight [g/24 h]	1.6 ± 0.2	1.3 ± 0.1

Metabolic parameters measured in 6–8 weeks old female wildtype and Tg6 mice fed with standard diet and kept in metabolic cages. Data is presented as mean ± s.e.m.; (*n* = 5 for each group of mice) and was analysed by unpaired Student’s t test with *p < 0.05 and **p < 0.01.

### FGF23, PTH, 1,25 (OH)_2_ vitamin D_3_, osteocalcin and deoxypyridinoline measurements

Plasma levels of Mouse FGF23 (intact) (Immutopics; 60–6800), Mouse/Rat C-terminal fragment of FGF23 (C-term. FGF23, Immutopics; 60–6300), PTH 1–84 (Immutopics; 60–2305) and osteocalcin (MicroVue Osteocalcin EIA, Quidel Corporation, Tecomedical AG, Sissach, Switzerland) were measured by ELISA, whereas plasma 1,25(OH)_2_ vitamin D_3_ was determined by radioimmunoassay (Immunodiagnostic System, Frankfurt am Main, Germany). Urinary deoxypyridinoline (DPD) were assessed with an enzymatic immunoassay kit (MicroVue DPD EIA, Quidel Corporation, Tecomedical AG, Sissach, Switzerland). All assays were performed according to the manufacturers protocols.

### Protein extractions and western blotting

Protein extractions and immunoblotting was performed as described before^21^. Briefly, mice kidney and Ileum were homogenized in ice cold re-suspension buffer (200 mM Mannitol, 80 mM Hepes, 41 mM KOH (pH 7.5) buffer supplemented with cocktail protease inhibitor (Complete; Roche Diagnostics, Basel, Switzerland). The homogenate was centrifuged at 2000 rpm for 20 min at 4 °C. The resulting total supernatant was further centrifuged at 41000 rpm for 1 hour at 4 °C in order to enrich the membrane proteins in the final pellet. Total protein concentration was measured using the Bio-Rad D_C_ protein Assay (Bio-Rad, Hercules, CA, USA). Fifty μg of either membrane or total proteins were solubilised in Laemmli buffer and separated on SDS-PAGE and transferred to (PVDF) polyvinylidene difluoride membranes (Immoblion-P, Millipore, Schaffhausen, Switzerland). After blocking nonspecific binding with 5% milk powder in Tris-buffered saline (TBS) containing 0.1% Tween-20 for 1 hour at room temperature, the blots were incubated overnight at 4 °C with primary antibodies against against α-Klotho (1:1000; TransGenic Inc.), VDR (1:1000; Santa Cruz), NaPi-IIa^22^ (1:2000), TRPV5^23^ (1:1000), Calbindin D28k (1:1000; SWANT, Marly, Switzerland), Cyp24a1 (1:1000; Protein Tech, Manchester, United Kingdom), goat anti-mouse C-terminal epitope of FGF23 (Immunotopics; 21–6320; 1:500), goat anti-mouse N-terminal epitope of FGF23 (Immunotopics; 21–6810, 1:500), or β-Actin (1:5000; Sigma-Aldrich). Upon 3 washes with TBS, membranes were incubated for 1 hour at room temperature with the appropriate (anti-rat, anti-rabbit, or anti-mouse) secondary antibodies either linked to horseradish peroxidase (HRP) or to Alkaline Phosphatase (1:5000; Promega AG, Dübendorf, Switzerland). After 3 washes with TBS, membranes were exposed to HRP substrate (Western Chemiluminescence HRP Substrate, Millipore, Schaffhausen, Switzerland) or Alkaline Phosphatase CDP-Star substrate (Roche) for 5 minutes. Chemiluminiscence was detected with a LAS-4000 camera system (Fujifilm). Densitometric analysis was performed using Advanced Image Data Analyzer (AIDA; Raytest).and the density of the proteins of interest was normalized to β-actin.

### Micro computer tomography (MicroCT)

The dissected femora were scanned with a micro-CT scanner (Quantum Fx, Perkin Elmer, Hopkinton, MA, USA) using a focal size of 5 mm, a tube voltage of 90 kV, a tube current of 100 μA and an isotropic voxel size of 10 μm. For calibration of bone data to bone mineral density (BMD) values a commercially available calcium hydroxyapatite phantom (QRM GmbH, Moehrendorf, Germany) was scanned using the same settings. All images were reconstructed and processed for bone analysis applying a commercially available software (Analyze 12.0, AnalyzeDirect, Inc., Overland Park, KS). A segment of 100 slices proximal to the distal femoral growth plate was analyzed starting where the epiphyseal cap structure completely disappeared. For BMD analysis the average grey level intensity was measured for the different calcium hydroxyapatite inserts of the phantom and a linear calibration was derived between grey level intensity and BMD.

### Statistics

Statistical significances were calculated by *t test* or one-way ANOVA (Bonferroni) as indicated. *p* < 0.05 was considered significant. Results are presented as means ± SEM.

## Results

### High haematocrit, iron deficiency, decreased liver hepcidin and splenomegaly in Tg6 mice

**Figure 1:**
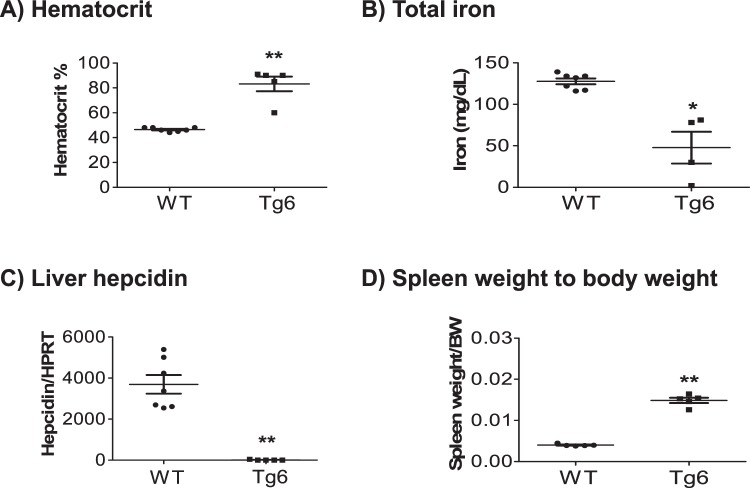
Over-expression of EPO induced high hematocrit, low iron, low liver hepcidin expression, and splenomegaly in Tg6 mice. Biological markers of EPO activity were assessed in 6–8 weeks old female Tg6 mice and their littermates: (**A**) hematocrit, (**B**) total plasma iron, (**C**) liver hepcidin mRNA expression, and (**D**) spleen weight normalized to body weight. Data are means ± s.e.m.; *n* = 5–7 mice for each genotype. Significance was determined by unpaired *t* test and indicated as *p < 0.05, **p < 0.01.

First, we verified the general characteristic hallmarks of 6–8 weeks Tg6 mice in comparison to wildtype (WT) littermates. As described previously^16,18,24^, Tg6 mice presented with extremely high hematocrit levels up to 90% (Fig. 1A) due to EPO-induced excessive erythrocytosis. Tg6 mice also developed iron deficiency (Fig. 1B) and accordingly, mRNA levels of liver hepcidin, the iron hormone^25^, was decreased (Fig. 1C). A marked splenomegaly was noted in Tg6 mice and was quantified as a ratio of spleen weight to body weight (Fig. 1D).

### Altered kidney function and mild albuminuria in 6–8 weeks old Tg6 mice

**Figure 2:**
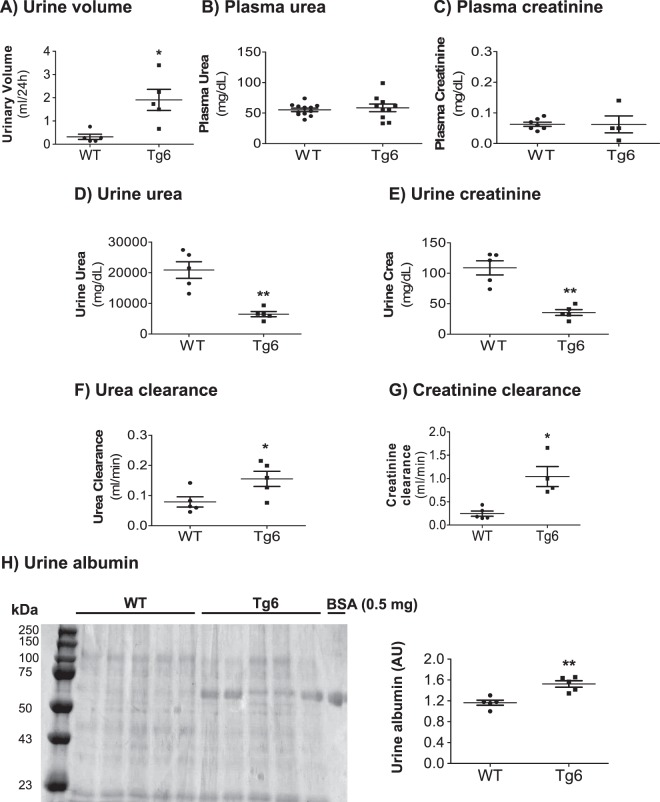
Markers of kidney function in Tg6 mice. Markers of kidney function were examined in 6–8 weeks old female Tg6 mice and their wildtype littermates: (**A**) urine volume per 24 hours, (**B**) plasma urea, **(C)** plasma creatinine, **(D)** urine urea, **(E)** urine creatinine, **(F)** urea clearance (ml/min), **(G**) creatinine clearance (ml/min), and **(H)** urine albumin excretion on a Coomassie blue stained gel with bovine serum albumin (BSA) as a positive control. Data are means ± s.e.m.; *n* = 5–12 for each group of mice. Significance was determined by unpaired *t* test and indicated as *p < 0.05, **p < 0.01.

Body weight and food intake was not different between Tg6 mice and littermates, but water intake (Table 1) and urinary volume (Fig. 2A) were both increased in Tg6 mice. Considering that 5 months old Tg6 mice had reduced kidney function^26^ and in view that FGF23 levels can be influenced by kidney function^2^, we first assessed markers of kidney function in young female Tg6 mice aged 6–8 weeks. Plasma urea and creatinine were similar in both genotypes (Fig. 2B,C). However, 24 hrs urine urea and creatinine were lower (Fig. 2D,E) and urea and creatinine clearance were elevated in Tg6 mice (Fig. 2F,G). Of note, higher urinary volume was paralleled by higher water intake in Tg6 mice whereas all other parameters examined were similar in both genotypes. Morevover, these young Tg6 mice exhibited mild albuminuria as evident from Coomassie blue band densitometry (Fig. 2H).

### Tg6 mice show mild hypercalciuria and increased FGF23 levels

**Figure 3:**
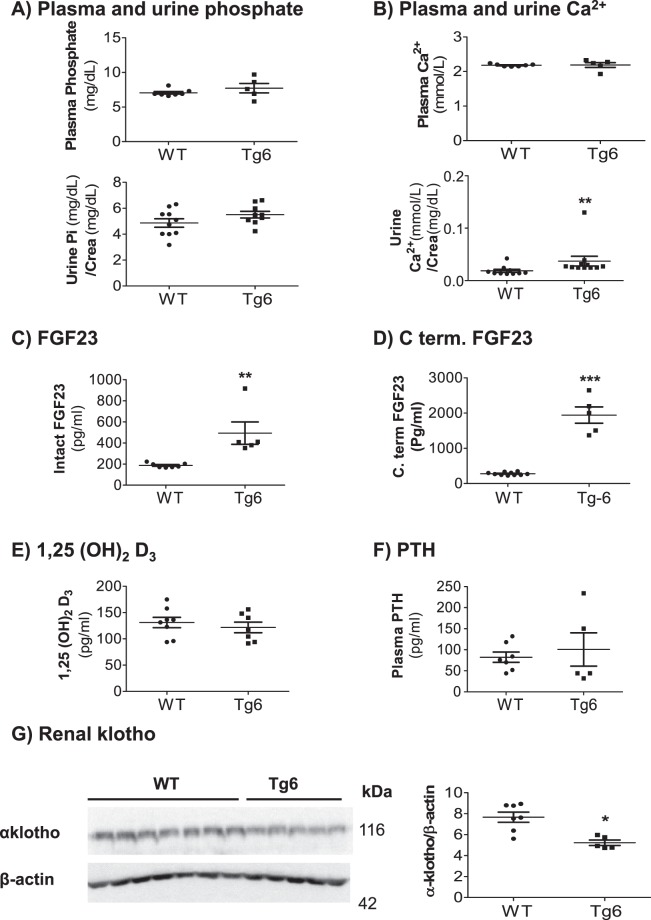
Higher urinary excretion of Ca^2+^ and elevated levels of both forms of FGF23 in Tg6 mice. (**A**,**B**) Plasma and urine concentrations of phosphate (Pi) and total calcium (Ca^2+^) in 6–8 weeks old female Tg6 mice and their littermates. (**C**) Plasma intact FGF23, (**D**) plasma C-terminal fragment of FGF23, **(E)**, plasma 1,25 (OH)_2_ vitamin D_3,_ and (**F**) plasma parathyroid hormone (PTH). (**G**) Renal α-klotho protein expression was detected by immunoblotting in kidney extracts of Tg6 mice and their controls. Membranes were reblotted for β-actin. The β-actin to α-klotho ratio is shown as a graph. Data are means ± s.e.m.; *n* = 5–10 for each group of mice. Significance was determined by unpaired *t* test and indicated as *p < 0.05, **p < 0.01.

To investigate whether chronic EPO over-production in Tg6 mice influenced mineral homeostasis, we examined plasma and urine phosphate as well as calcium levels in Tg6 mice and their littermates. As shown in Fig. 3A,B, both phosphate and calcium were similar in plasma between groups but urinary calcium excretion was mildly elevated in Tg6 mice versus the control group. Phosphate excretion remained unchanged (Fig. 3A). Next, we investigated the plasma levels of endocrine key regulators of mineral metabolism. Chronically elevated EPO synthesis enhanced plasma levels of the intact form of FGF23 (Fig. 3C) and of the C-terminal fragment of FGF23 (Fig. 3D), whereas 1, 25(OH)_2_ vitamin D_3_ and PTH levels were unaffected (Fig. 3E,F). The renal expression of the obligatory FGF23 co-receptor, α-klotho was decreased (Fig. 3G).

### Liver and spleen of Tg6 mice ectopically express FGF23

**Figure 4:**
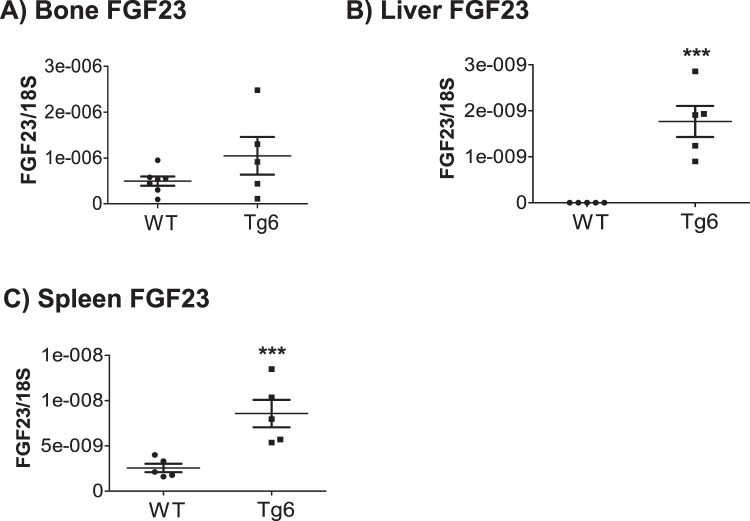
Chronic EPO over-expression induced FGF23 expression in spleen and liver of Tg6 mice. FGF23 mRNA levels were examined in (**A**) femur, **(B)** liver, and (**C**) spleen of 6–8 weeks old female Tg6 mice versus control. Expression levels of FGF23 were normalized to ribosomal 18 s. Data are means ± s.e.m.; *n* = 5–7 for each group of mice. Significance was determined by unpaired *t* test and indicated ***p < 0.001.

Next we sought to identify sources of elevated FGF23. Previously, we and others had shown that EPO induced FGF23 expression mainly in bone marrow^5,9–11^. Therefore, we examined FGF23 mRNA expression in bone and bone marrow and also in liver and spleen of Tg6 and control mice. Notably, bone marrow cells of Tg6 mice did not express any detectable FGF23 mRNA (data not shown). As shown in Fig. 4A, bone FGF23 mRNA expression levels did not differ between Tg6 mice and controls. Also Galnt3 and Phex, two factors involved in bone and bone marrow FGF23 gylcosylation and degradation, respectively, were unchanged (Supplementary Fig. 1). In contrast, liver and spleen of Tg6 mice expressed robustly FGF23 mRNA whereas no FGF23 mRNA was detected in the corresponding wild type control organs (Fig. 4B,C). We also attempted to detect FGF23 protein in spleen and liver (Supplementary Figs 5, 6) but failed to detect FGF23 protein whereas it was readily detectable in bone extracts prepared from Phex mutant mice^27^.

### Normal 1,25 (OH)_2_ vitamin D_3_ metabolism in Tg6 mice

**Figure 5:**
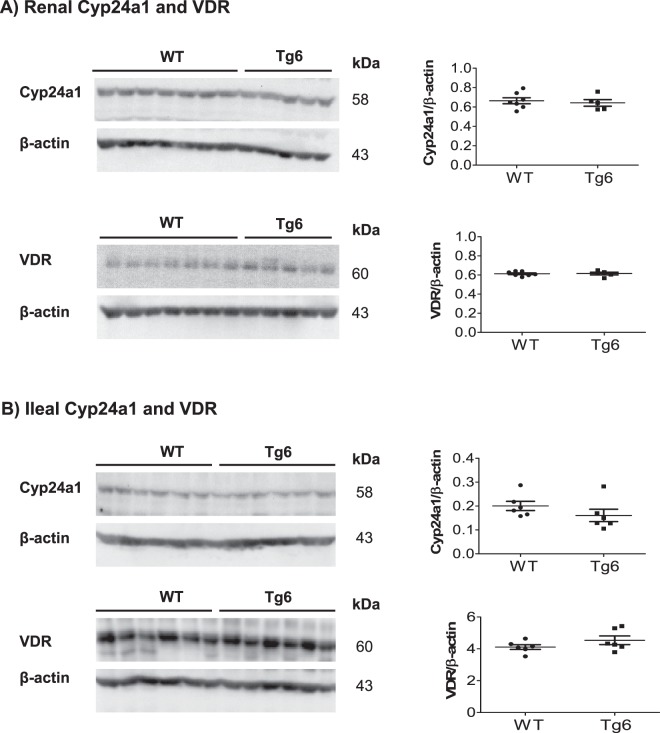
Normal 1,25 (OH)_2_ vitamin D_3_ metabolism in Tg6 mice. Expression of the enzymes metabolizing 1,25 (OH)_2_ vitamin D_3_ and the vitamin D receptor (VDR) was examined by immunoblotting in kidney and ileum of 6–8 weeks old female Tg6 mice and their controls. (**A**) Renal expression of Cyp24a1 and the vitamin D receptor (VDR) and (**B**) ileal expression of Cyp24a1 and the VDR. For immunoblots, all membranes were stripped and re-probed for β-actin. Graphs show the ratio of the protein of interest over β-actin. Data are means ± s.e.m.; *n* = 5 for each group of mice.

Since FGF23 modulates the expression and activity of the enzymes synthesizing and degrading 1,25 (OH)_2_ vitamin D_3_ we examined their expression along with the vitamin D receptor (VDR). The protein abundance of the 24-hydroxylase (Cyp24a1) and VDR in kidney (Fig. 5A) and ileum (Fig. 5B) were similar in both genotypes. However, despite of the constant plasma level of 1,25 (OH)_2_ vitamin D_3_ between the groups (Fig. 3F), kidney α_1_-hydroxylase (Cyp27b1) mRNA expression was less in Tg6 mice kidney (Supplementary Fig. 2). Ileum Cyp27b1 expression did not change between the Tg6 and control groups (Supplementary Fig. 3) as well as ileum VDR mRNA levels were similar in both mouse groups.

### Decreased expression of renal key proteins of phosphate and calcium transport in Tg6 mice

**Figure 6:**
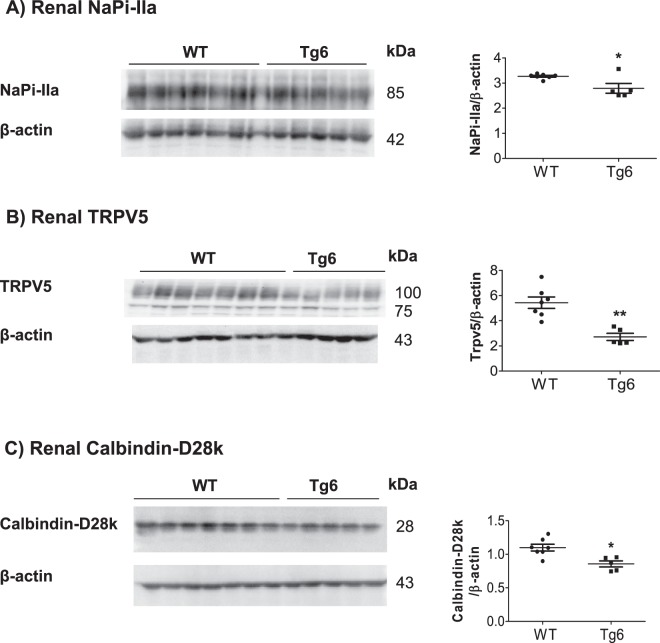
EPO over-expression reduced expression of renal proteins involved in Ca^2+^ and phosphate transport in Tg6 mice. The protein expression of the (**A**) sodium phosphate co-transporter NaPi-IIa, (**B**) the epithelial Ca^2+^ channel TRPV5, and (**C**) the cytoplasmic Ca^2+^ -binding protein Calbindin-D28k protein was tested by immunoblotting of kidney extracts from 6–8 weeks old female Tg6 mice and their littermate controls. All membranes were stripped and reprobed for β-actin. Graphs show the ratio of the protein of interest over β-actin. Data are means ± s.e.m.; *n* = 5 for each group of mice. Significance was determined by unpaired *t* test and indicated as *p < 0.05 and **p < 0.01.

We next examined the renal expression of key proteins of tubular phosphate and calcium reabsorption along the nephron. The abundance of the main proximal tubular sodium phosphate co-transporter, NaPi-IIa, was significantly reduced in Tg6 mice (Fig. 6A). Similarly, the protein abundance of the TRPV5 calcium channel and the calcium-buffering protein calbindin-D28k, expressed in the distal convoluted tubule and connecting tubule, was significantly decreased consistent with elevated urinary calcium excretion (Fig. 6B,C). Semi-quantitative real-time PCR for the ileum sodium phosphate co-transporter, NaPi-IIb, revealed decreased expression levels in Tg6 mice (Supplementary Fig. 4).

### Tg6 mice exhibited reduced bone mineral density

**Figure 7:**
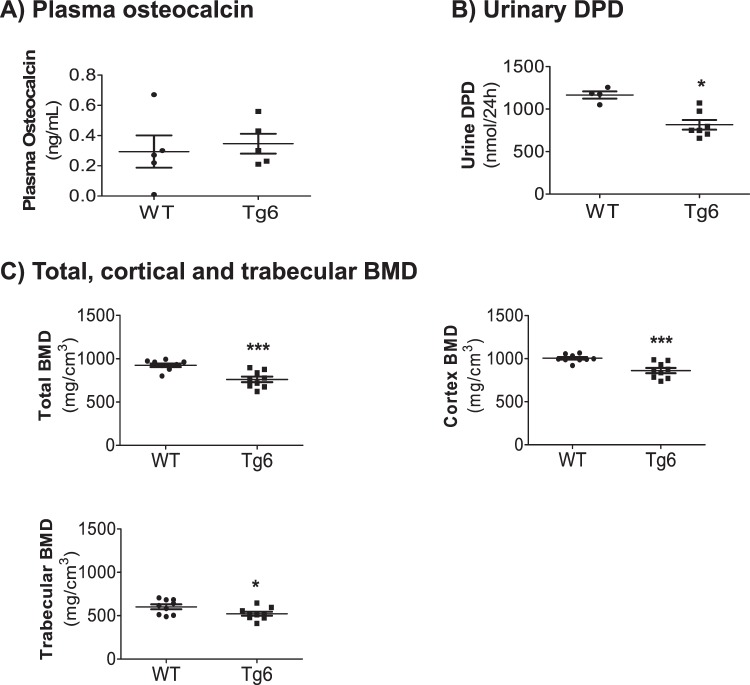
Bone phenotype of 6–8 weeks old female Tg6 mice. The bone phenotype of 6–8 weeks old female Tg6 mice and their wildtype littermates was examined by assessment of bone turnover markers in plasma and urine. (**A**) Plasma osteocalcin and (**B**) urinary DPD excretion/24 hrs. (**C**) Total, cortical and trabecular bone mineral density (BMD).

To clarify if impaired calcium homeostasis in Tg6 mice parallels reduced bone mineral density and turnover as reported previously in 5 month old Tg6 mice^17,28^, we quantified the level of the bone formation marker osteocalcin in plasma as well as urinary excretion of the bone degradation marker deoxypyridinoline (DPD)^29,30^. As shown in Fig. 7A, no difference was detectable in the plasma level of osteocalcin between the groups. In contrast, urinary excretion of DPD was reduced in the urine of Tg6 mice (Fig. 7B).

**Figure 8:**
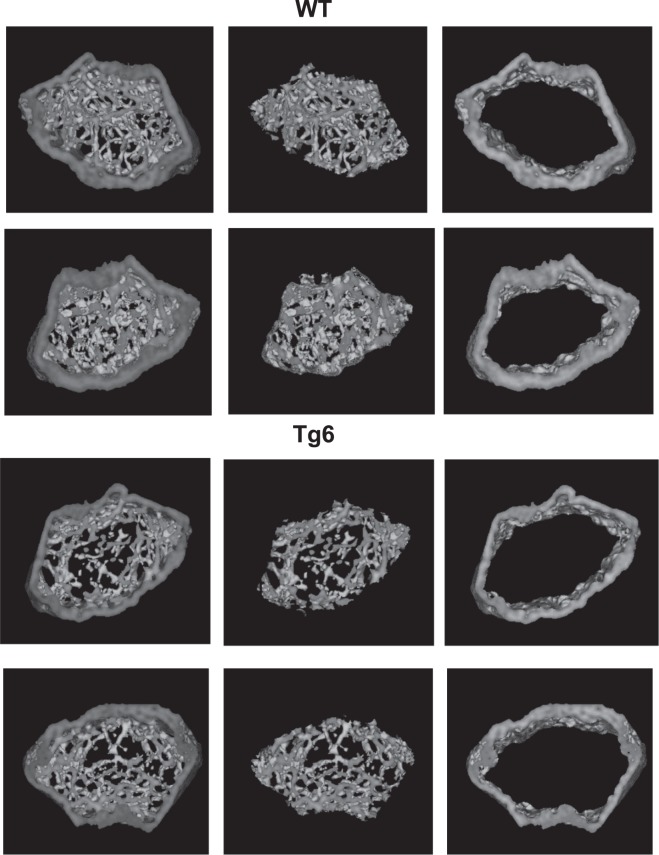
Bone phenotype of 6–8 weeks old female Tg6 mice. The bone phenotype of 6–8 weeks old female Tg6 mice and their wildtype littermates was examined by μCT. Representative cross-sections microCT images of distal femora of WT versus Tg6 are shown. Dark grey area indicates mineralized bone. Data are means ± s.e.m.; *n* = 5–9 for each group of mice. Significance was determined by unpaired *t* test and indicated as *p < 0.05 and ***p < 0.001.

Finally, microCT of femora was used to assess bone mineral density and architecture of Tg6 mice. We found that total, cortical and trabecular bone mineral density were lower in femora from Tg6 mice as compared to their littermates (Fig. 7C, Fig. 8). For the trabecular bone, total volume (TV) and trabecular number (Tb.N) were increased in Tg6 mice whereas connectivity density (Conn.D) was increased. All other parameters were not different between groups (Table 2).

**Table 2:** MicroCT bone measurements.

	WT	Tg6
Total volume (TV) (mm^3^)	2.97 ± 0.03	3.12 ± 0.04*
Bone volume (BV) (mm^3^)	1.15 ± 0.05	1.28 ± 0.04
BV/TV (%)	38.9 ± 1.5	41.2 ± 1.0
Trabecular number Tb.N (1/mm)	2.09 ± 0.21	2.83 ± 0.22*
Trabecular thickness Tb.Th (mm)	0.07 ± 0.01	0.07 ± 0.01
Trabecular separation Tb.Sp (mm)	0.20 ± 0.01	0.17 ± 0.01*
Connectivity density Conn.D (1/mm^3^)	51.5 ± 8.9	111.0 ± 13.4**

Parameters from microCT analysis of femora of 6–8 weeks old female Tg6 mice and littermates. Total bone volume (TV), bone volume (BV), trabecular bone fraction (BV/TV), thickness of the trabeculae (Tb.Th), trabeculae number (Tb.N), connectivity density (Conn.D), and trabecular separation (Tb.Sp) values are given for Tg6 and control groups. Data is presented as mean ± s.e.m.; *n* = 9 for each group of mice, data were analysed by unpaired Student’s t-test with *p < 0.05 and **p < 0.01.

## Discussion

In the present paper we analyzed the effects of a chronic elevation of EPO on FGF23 and mineral homeostasis in a well-establisched mouse model. We first ascertained hallmarks of this mouse model by demonstrating a highly increased hematocrit, low total iron, highly reduced liver hepcidin levels, and splenomegaly consistent with the biological effects of chronically elevated EPO^13,14,25^. Next we examined markers of renal function as such a decrease *per se* can be associated with higher FGF23. We choose young mice, i.e. 6–8 weeks old, as decreased kidney function has been described in older mice (5 months old)^26^. Tg6 mice showed signs of elevated glomerular filtration as evident from higher urea and creatinine clearance but with normal urea and creatinine plasma levels. Indeed, erythropoietin has been shown to stimulate GFR and protect glomerular functions^31,32^. Tg6 mice had also mild albuminuria. The combination of polycythemia (erythrocytosis), hypertension, and mild albuminuria with normal glomerular filtration rate and elevated fractional filtration has been described as High Altitude Renal Syndrome (HARS) in people adapted to living in high altitude in the Andes^33^. The pathogenesis of HARS is not fully understood but our findings in the Tg6 mice are reminiscent of some of the main features. Thus, our data cannot fully exclude a mild damage of kidney function that could contribute to higher FGF23 levels. Increased GFR may precede a loss of renal function as observed in diabetic nephropathy.

Acutely increased EPO stimulates FGF23 with both intact and C-terminal FGF23 being elevated^8–10,34^. Our data from the Tg6 mice are also consistent with this stimulatory effect in conditions of chronically elevated EPO. Both forms of FGF23 were higher in Tg6 mice and paralleled by downregulation of NaPi-IIa, α-klotho, and Cyp27b1 in kidney. However, no renal phosphaturia or lower 1,25 (OH)_2_ vitamin D_3_ were detectable, while we had previously found lower 1,25 (OH)_2_ vitamin D_3_ during acute treatment WT mice with EPO^10^. The absence of phosphaturia in the face of downregulated NaPiIIa may be explained by a lower delivery of phosphate to the proximal tubule due to a lower plasma volume available for filtration. Also, a partial resistance to FGF23 may prevent a stronger downregulation of NaPi-IIa and a suppression of 1,25 (OH)_2_ vitamin D_3_. We have previously found a similar resistance in an animal model of autosomal dominant polycystic kidney disease (ADPKD)^35^. In contrast to acute EPO injections where EPO mostly stimulated FGF23 production by erythroid cells in the bone marrow, we did not detect evidence for a stimulatory effect of chronically elevated EPO in bone marrow. We found FGF23 mRNA but not protein in spleen and liver suggesting that both organs might be sources of elevated FGF23 under conditions of long-term elevated EPO levels. Spleen has been previously identified as a source of FGF23 in lipopolysaccharide (LPS) induced inflammation and expresses also the FGF23 co-receptor α-klotho^36,37^. Likewise, liver has been suggested to be a source of elevated FGF23 in ADPKD^38^. Elevated C-terminal FGF23 may be caused, at least in part, by depletion of iron stores due to excessive hematopoiesis. Iron depletion has been shown to increase the C-terminal fragment in other mouse models^39,40^.

Tg6 mice suffered from a mild but clear disturbance of mineral metabolism. Tg6 mice had mild hypercalciuria which is probably caused by the downregulation of TRPV5 calcium channels and calbindin D28k, both expressed in the distal convoluted tubule and connecting tubule, the last nephron segment capable of calcium reabsorption^41^. The dysregulation of TRPV5 and calbindin D28k cannot be easily explained by altered levels of PTH, 1,25-(OH)_2_ vitamin D_3_, or VDR. However, α-klotho levels in kidney were reduced and α-klotho stabilizes TRPV5 at the luminal membrane and maintains its activity^3,42,43^. Whether the function(s) of α-klotho involve enzymatic activity or not is still debated, the recently published structure of α-klotho, however, is incompatible with the previously proposed enzymatic activity^44^. Nevertheless, reduced α-klotho levels might be responsible for reduced TRPV5 activity but other α-klotho independent mechanisms could not be excluded at this stage. Calbindin D28k downregulation may be the consequence of reduced apical calcium entry. The cause of lower renal α-klotho expression may be the elevation of FGF23. However, renal α-klotho expression is very sensitive to renal injury and α-klotho falls very early in the course of renal disease^2,45^. Thus, the mild renal impairment observed in the Tg6 mice may also contribute to the lower α-klotho levels even though hypercalciuria is not a common finding in early CKD suggesting that the hypercalcuria is not only caused by renal impairment.

Tg6 mice had also changes in their bone mineral density as part of their disturbed mineral homeostasis. In a previous study a lower trabecular bone mass was observed in 12 weeks old Tg6 mice in combination with a 25% higher osteoclast number when compared to wildtype control animals – suggesting that EPO directly stimulates osteoclast activity leading to an inappropriate hyperabsorption of bone^17,46^. In contrast, in this study we see more but less mineralized tissue in 6–8 weeks old Tg6 mice compared to their wildtype littermates. The lower bone mineral density came along with an increased urinary excretion of calcium in Tg6 mice, suggesting that the impaired mineralisation might be triggered or enhanced by an increased loss of calcium. A reduced urinary excretion of DPD (bone resorption marker) was observed in Tg6 mice which might indicate that at the age of 6–8 weeks their bone resorption activity is lower than in their wildtype littermates and that they might actively try to compensate the loss of bone quality at this young age.

The impaired bone mineralization already observed at the age of 6–8 weeks in the Tg6 mice is possibly maintained by a combination of high EPO and high FGF23 suppressing a possible counterregulation by PTH.

We noted also that the expression of the intestinal NaPi-IIb phosphate co-transporter was reduced in Tg6 mice. We have previously shown that this transporter in situation of low phosphate availability is important to protect bone from demineralization^47^.

This study has some limitations. The high haematocrit may cause alterations in kidney function as well as in bone mineralisation and endocrine regulation. However, this is inherent to models of chronically elevated EPO levels. Second, we studied only female mice. Third, our results do not rule out that other factors than FGF23 are responsible for the alterations in mineral metabolism and bone architecture.

Taken together, our Tg6 mice that represent an established model for chronic EPO elevation in plasma, show profound signs of disturbed mineral homeostasis including inappropriately high FGF23 levels which might stem from aberrant production by liver and spleen, low renal α-klotho levels, mild urinary calcium wasting, reduced expression of the renal and intestinal phosphate transporters NaPi-IIa and NaPi-IIb and importantly reduced trabecular bone mineral density which is most likely depicting an impaired bone mineralization at this age. Thus, reduced bone mineralization in patients with CKD and EPO therapy may require further investigation.

## Supplementary information

supplementary data and full size blots

## Data Availability

All protocols and raw data are available upon request.
