# Supplementary material for: Elevated FGF23 and disordered renal mineral handling with reduced bone mineralization in chronically erythropoietin over-expressing transgenic mice
Source: Sci Rep. 2019 Oct 18;9:14989. doi: 10.1038/s41598-019-51577-z (PMC6802194; doi:10.1038/s41598-019-51577-z)
Supplement: Supplementary file 1 — supplementary data and full size blots [file 41598_2019_51577_MOESM1_ESM.pdf]

SUPPLEMENTARY DATA for

Elevated FGF23 and disordered renal mineral handling with reduced bone mineralization in chronically erythropoietin over-expressing transgenic mice

Arezoo Daryadel[1,2,3], Luciano Natale[1], Petra Seebeck[2,4], Carla Bettoni[1,2,3], Udo Schnitzbauer[1,2,3], Max Gassmann[2,5,6], Carsten A. Wagner[1,2,3]

[1]Institute of Physiology, University of Zurich, Zurich, Switzerland

[2]Zurich Center for Integrative Human Physiology (ZIHP), University of Zurich, Zurich, Switzerland

[3]National Centre for Competence in Research NCCR “Kidney.CH”, Switzerland

[4]Zurich Integrative Rodent Physiology (ZIRP), University of Zurich, Switzerland

[5]Institute of Veterinary Physiology, University of Zurich, Switzerland

[6]Universidad Peruana Cayetano Heredia (UPCH), Lima, Peru

This file contains:

- **Additional data**
- **Original full size blots**

Supplementary Figures.

Supplementary Figure 1.
Relative mRNA expression of bone and bone marrow Galnt3 and Phex in 6 – 8 weeks old female Tg6 mice and littermates. Galnt3 and Phex mRNA values were normalized to ribosomal 18s (rRNA). Data are means ± s.e.m.; *n*=6-8 for each group of mice and was analysed by unpaired Student's t test with $*p<0.05$.

Supplementary Figure 2. Relative mRNA expression of renal Cyp27b1 in 6 – 8 week old female Tg6 mice and littermates. Cyp27b1 mRNA values were normalized to ribosomal 18s (rRNA). Data are means ± s.e.m.; *n*=6-8 for each group of mice.

Supplementary Figure 3. Relative mRNA expression of ileal VDR and Cyp27b1 in 6 – 8 week old female Tg6 mice and littermates. VDR and Cyp27b1 mRNA values were normalized to ribosomal 18s (rRNA).. Data are means ± s.e.m.; *n*=6-8 for each group of mice.

Supplementary Figure 4. Relative mRNA expression of the ileal sodium phosphate co-transporter NaPi-IIb in 6 – 8 weeks old female Tg6 mice and littermates. NaPi-IIb mRNA values were normalized to ribosomal 18s (rRNA). Data are mean ± s.e.m.; *n*=8 for each group of mice and was analysed by unpaired Student's t test with $*p<0.05$.

Supplementary Figure 5: Immunoblotting of spleen total homogenate prepared from 6-8 week old female WT and Tg6 littermate mice. Bone extract from Phex mutant mice[26] was loaded as positive control for FGF23 protein expression. Blots were probed with antibodies direct either against a C-terminal or N-terminal epitope of FGF23. FGF23 protein species detected in Phex bone samples are indicated by red arrows. A shorter and a longer exposure of both blots are shown. All blots were reprobed for β-actin to control for equal loading of spleen samples.

Supplementary Figure 6: Immunoblotting of liver total homogenate prepared from 6-8 week old female WT and Tg6 littermate mice. Bone extract from Phex mutant mice[26] was loaded as positive control for FGF23 protein expression. Blots were probed with

antibodies direct either against a C-terminal or N-terminal epitope of FGF23. FGF23 protein species detected in Phex bone samples are indicated by red arrows. All blots were reprobed for β-actin to control for equal loading of spleen samples.

Supplementary table 1: List of primers and probes used for semi-quantitative real-time RT-PCR.

Supplementary data

Supplementary figure 1

Bone marrow

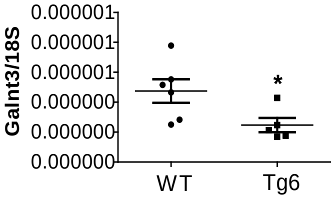

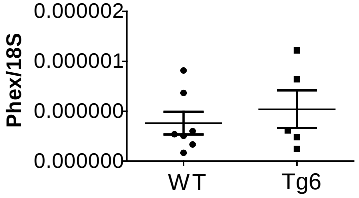

Bone

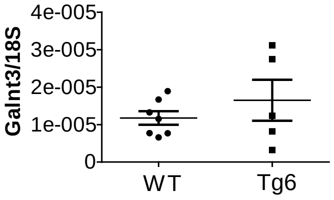

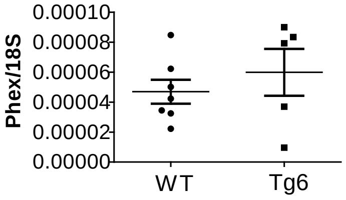

Supplementary figure 2

Kidney

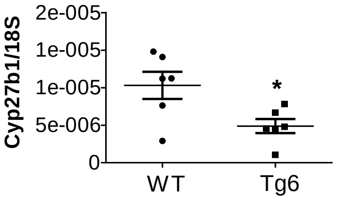

Supplementary figure 3

Ileum

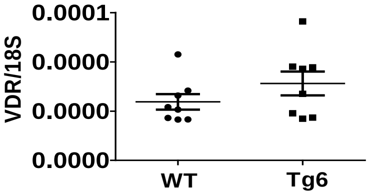

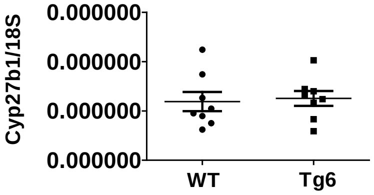

Supplementary figure 4

Ileum

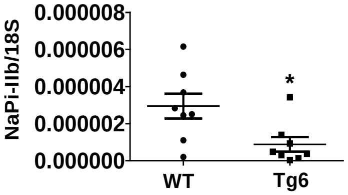

Supplementary figure 5

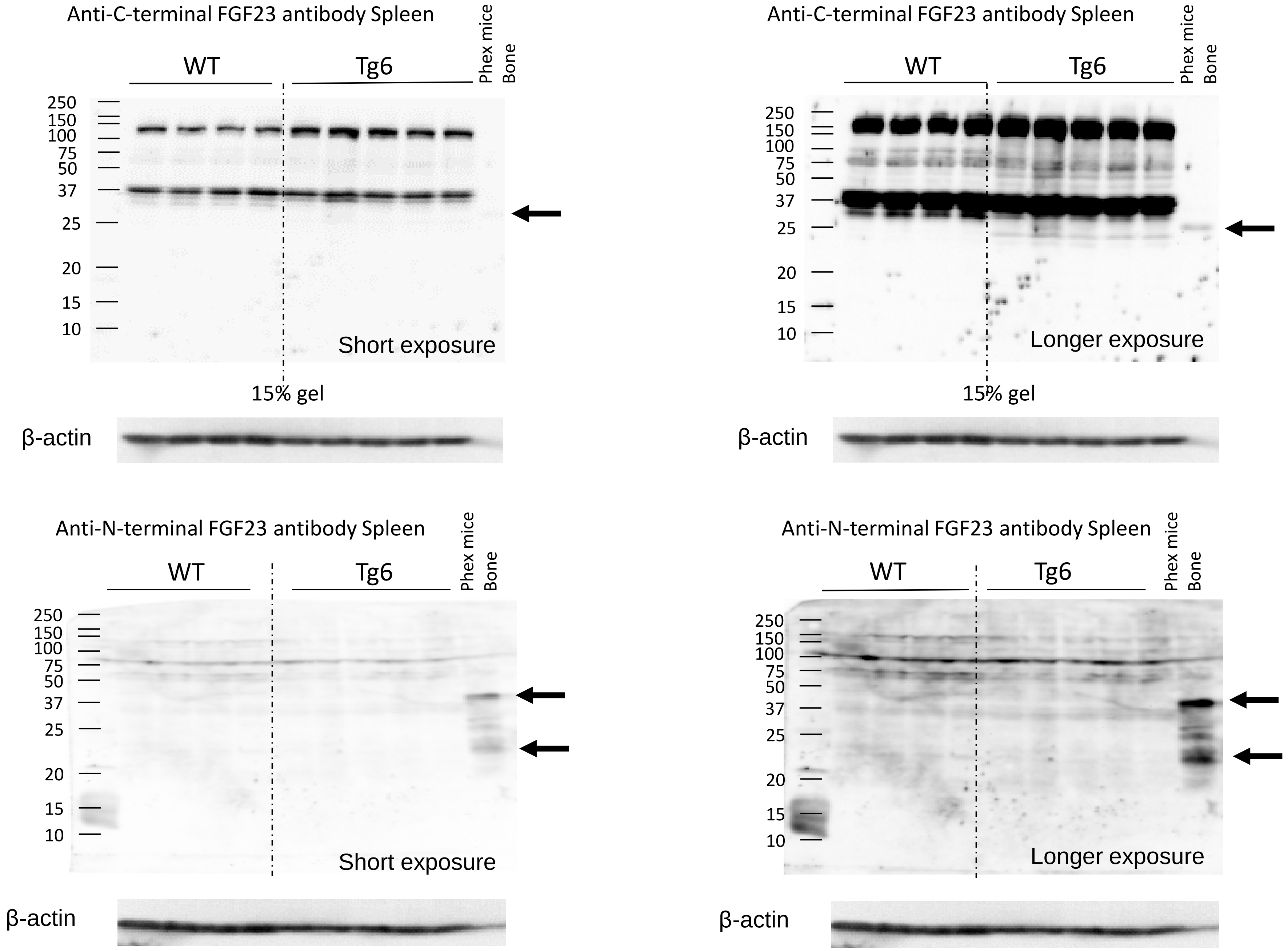

Supplementary figure 6

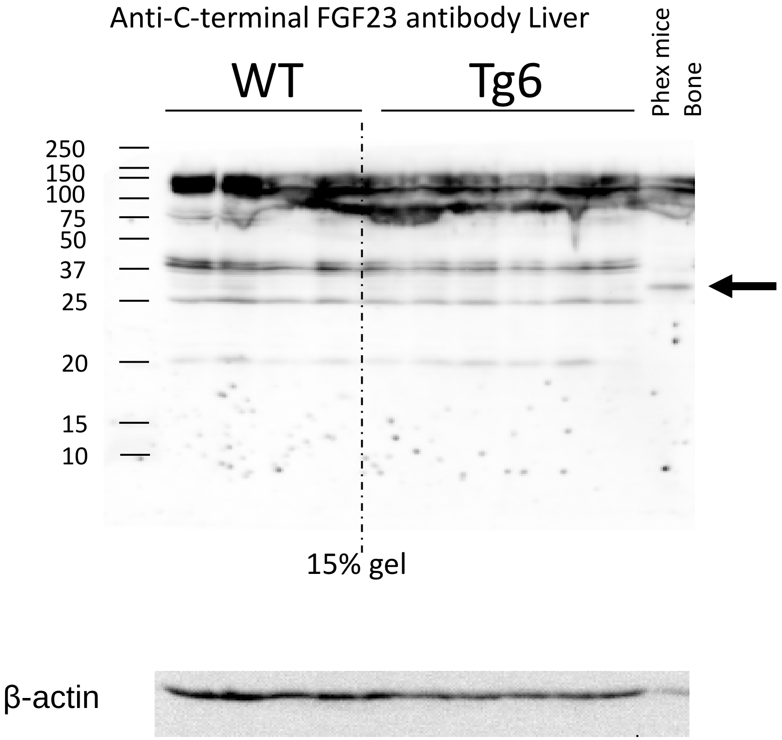

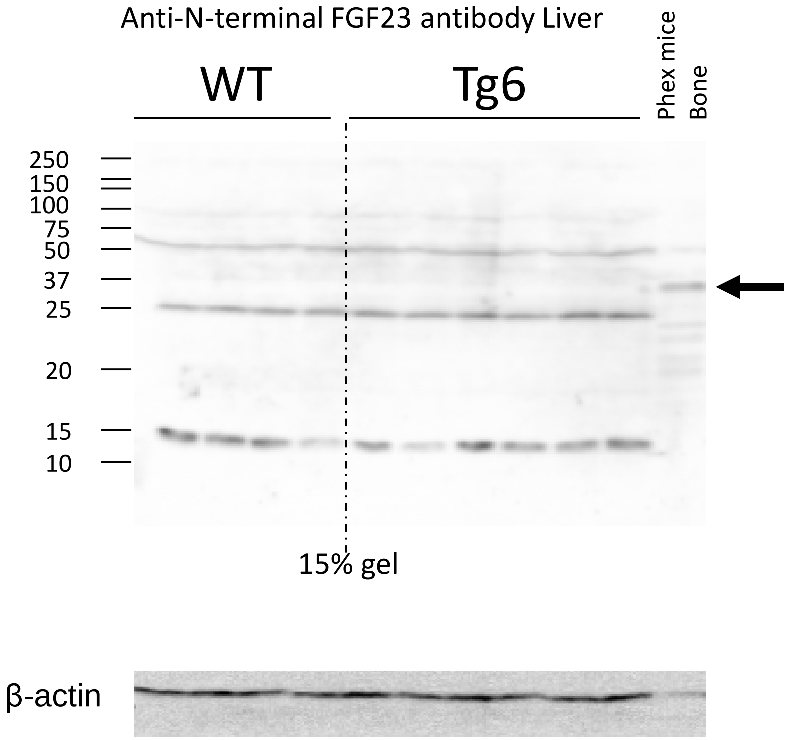

Full size immunoblots

Figure 2H (Urine coomasie blue)

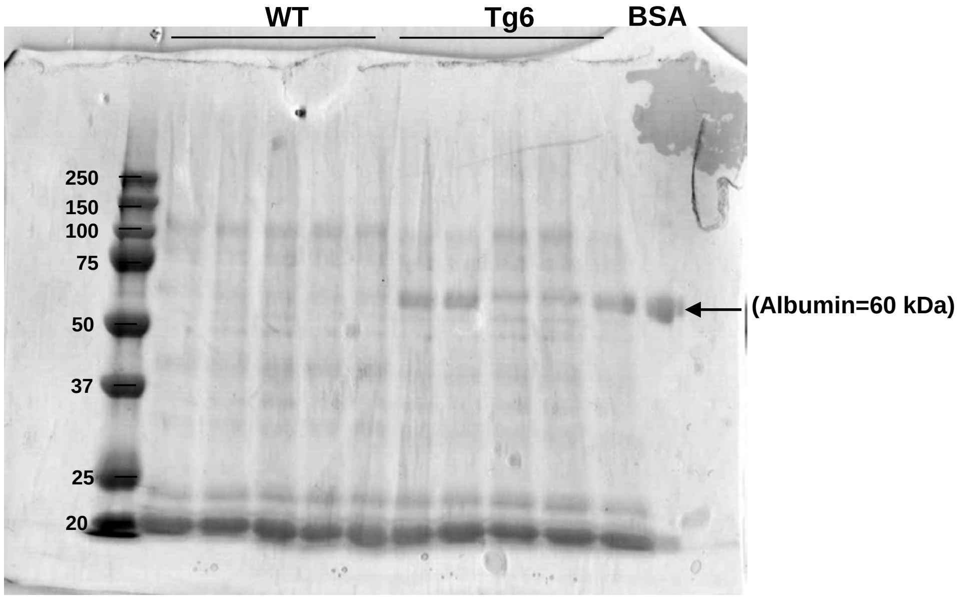

Figure 3F (Renal Klotho)

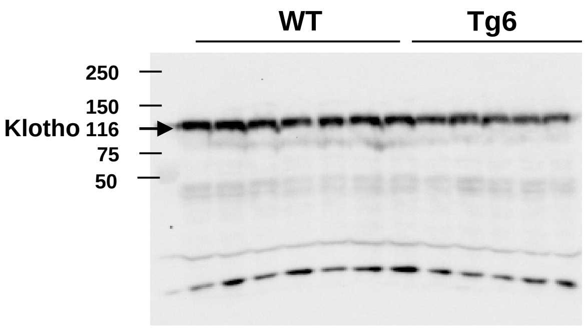

Figure 5A (Renal Cyp24a1)

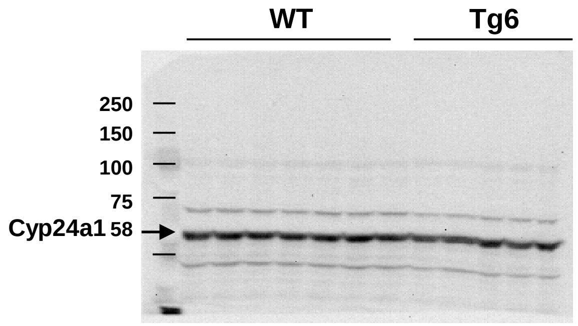

10% gel

Figure 5A (Renal VDR)

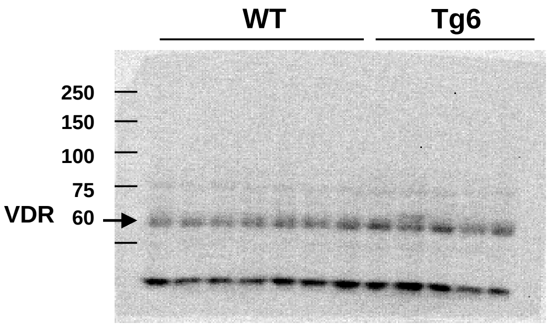

10% gel

Figure 5B (Ileal Cyp24a1)

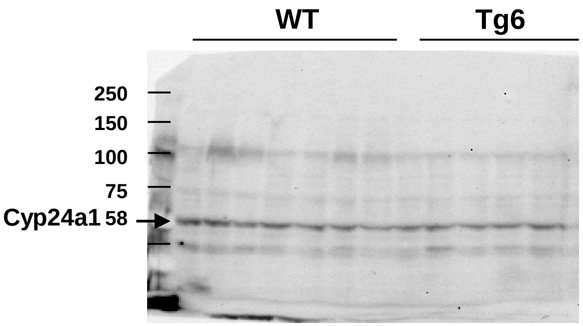

10% gel

Figure 5B (Ileal VDR)

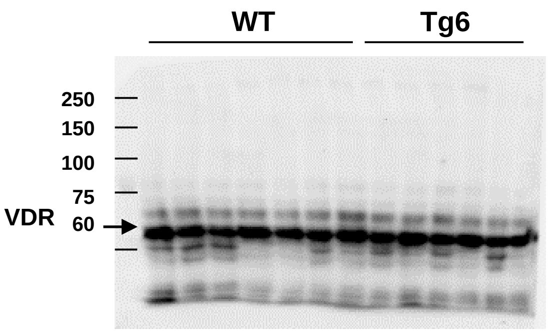

10% gel

Figure 6A (Renal NaPi-IIa)

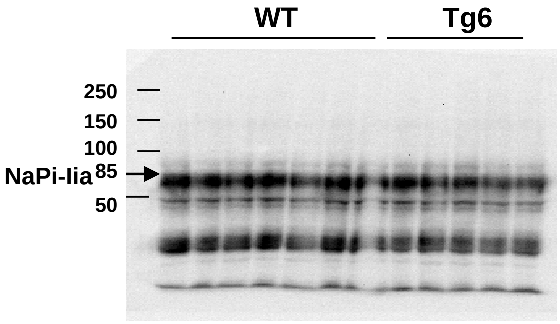

8% gel

Figure 6B (Renal TRPV5)

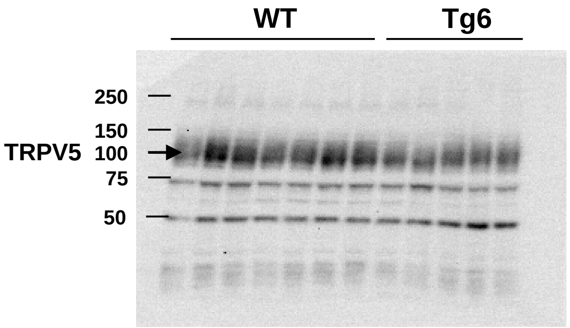

8% gel

Figure 6C (Renal Calbindin-D28k)

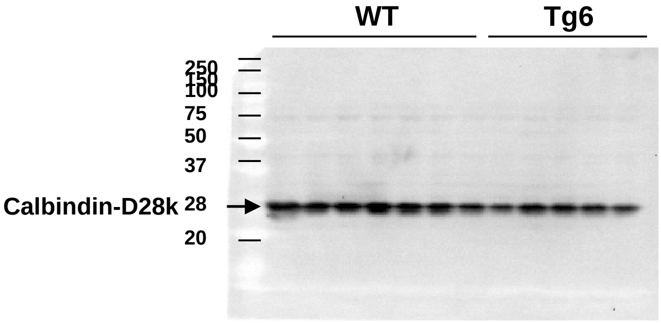

12% gel
